# Association between liver function and metabolic syndrome in Chinese men and women

**DOI:** 10.1038/srep44844

**Published:** 2017-03-20

**Authors:** Sen Wang, Jie Zhang, Li Zhu, Linlin Song, Zhaowei Meng, Qiang Jia, Xue Li, Na Liu, Tianpeng Hu, Pingping Zhou, Qing Zhang, Li Liu, Kun Song, Qiyu Jia

**Affiliations:** 1 Department of Nuclear Medicine, Tianjin Medical University General Hospital, Tianjin, P.R. China; 2 Section of Ultrasound and Interventional Therapy in Department of Surgery, Tianjin Medical University General Hospital, Tianjin, P.R. China; 3 Department of Ultrasound, Tianjin Medical University General Hospital, Tianjin, P.R. China; 4 Department of Health Management, Tianjin Medical University General Hospital, Tianjin, P.R. China

## Abstract

Metabolic syndrome (MS) could be associated with liver function. Our study aimed to investigate the association between liver function and MS in a large cohort of Chinese men and women. We enrolled 32,768 ostensibly healthy participants. The associations between liver function and MS of both genders were analyzed separately after dividing total bilirubin (TBIL), gamma glutamyltransferase (GGT), alanine aminotransferase (ALT) into quartiles. Young males had significantly higher MS prevalence than females, yet after menopause, females had higher MS prevalence. We used TBIL, GGT and ALT quartiles as categorical variables in binary logistic regression models. Significantly decreased MS risks were demonstrated in TBIL quartiles 2 to 4 for males, and quartiles 3 to 4 for females. As to GGT and ALT, significantly increased MS risks were shown in high quartiles for both genders. Aging also resulted in significantly higher MS risks in both genders except for young females. This study displayed close associations between liver function and MS, which were influenced by gender and age. A high TBIL level had protective effect against MS, while high GGT and ALT levels were risk factors for MS. It is meaningful that liver function is used as clinical risk predictors for MS.

Metabolic syndrome (MS) is a cluster of disorders, which includes obesity, hyperglycemia, hypertriglyceridemia, hypertension, and decreased high-density lipoprotein cholesterol (HDL). In 2009, a consensus criterion about the definition of MS was reached by a joint statement from the International Diabetes Federation Task Force on Epidemiology and Prevention; National Heart, Lung, and Blood Institute; American Heart Association; World Heart Federation; International Atherosclerosis Society; and International Association for the Study of Obesity^1^. Briefly, MS is diagnosed when three of the following five criteria suffice: increased waist circumference (WC), elevated triglycerides (TG), reduced HDL, elevated blood pressure (BP), and elevated fasting glucose (FG). People with MS are a susceptible population of many diseases, like type 2 diabetes and cardiovascular diseases^2^,^3^. Sex and age have great influence on the development of MS. MS prevalence increases during aging, but before menopause men have significantly higher MS risk than females, after menopause females have higher MS risk than males^4^,^5^,^6^,^7^,^8^,^9^.

Several studies have indicated that liver function, in particular, total bilirubin (TBIL), gamma glutamyltransferase (GGT), alanine aminotransferase (ALT), was closely correlated with MS. For example, Perera *et al*.^10^ examined elevated liver marker concentrations among Thai adults and suggested that elevated liver enzymes were related with MS risk. A study by Wei *et al*.^11^ reported that the transaminitis in obese children was associated with the components of MS from a UK-based obesity clinic. In the study by Zhang *et al*.^12^, data from a large sample with 6,268 MS subjects and 6,330 healthy controls were analyzed, which displayed significant associations between MS risk and liver enzymes. However, some other studies showed obvious inconsistency. For instance, the study by Monica *et al*.^13^ showed that only raised GGT was associated with all the features of MS among all the liver function parameters. A study in a Japanese population reported a negative association between TBIL and MS only in men but not in women^14^.

Therefore, the objective of this cross-sectional study was to investigate correlations between liver function and MS with emphasized focuses on differences generated from age and gender in a representative sample of Tianjin municipality population.

## Results

### Characteristics of the participants in different genders

The participants in opposite sex showed significant differences among the parameters except for low-density lipoprotein (LDL) (Table 1). On the whole, males were younger than females. Body mass index (BMI), WC, systolic blood pressure (SBP), diastolic blood pressure (DBP), TG, blood urea nitrogen (BUN), uric acid (UA), creatinine (Cr) and FG in males were significantly higher than in females. Yet HDL and total cholesterol (TC) in males were lower than those values in females. The indices representing hepatic function (TBIL, GGT and ALT) were significantly higher in males than those in females.

### Characteristics of liver function in different gender

TBIL quartiles 1 to 4 in males were defined according to the followings cut-off values: 9.90 μmol/L, 12.90 μmol/L and 16.80 μmol/L; for females, 8.00 μmol/L, 10.30 μmol/L, 13.40 μmol. GGT quartiles 1 to 4 in males were defined by the cut-off values of 20.00U/L, 30.00U/L and 48.00U/L; for females, 11.00U/L, 15.00U/L and 21.00U/L. For males, ALT quartiles 1 to 4 were according to 17.00U/L, 24.00U/L and 34.00U/L; for females, 13.00U/L, 16.00U/L and 22.00U/L.

In our study, we found that of the population, males had higher percentage in the highest quartile of TBIL than female before menopause, yet a reverse difference was displayed after menopause (Table 2). The similar results were also displayed in the highest quartiles of GGT and ALT (Tables 3 and 4).

### Prevalence of MS in different genders

**Figure 1:**
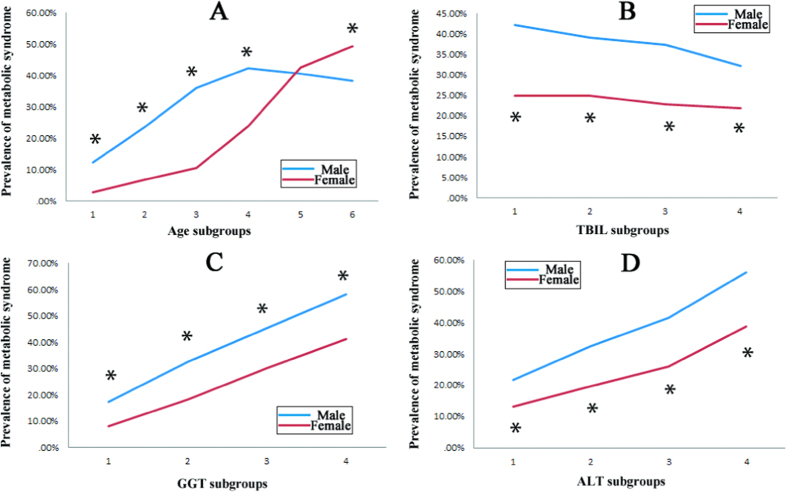
Prevalence of metabolic syndrome in different age and liver function subgroups. (**A**) Prevalence of metabolic syndrome in different age subgroups. Age subgroups 1 to 6 referred to the followings: age ≤ 25 years, 25 years < age ≤ 35 years, 35 years < age ≤ 45 years, 45 years < age ≤ 55 years, 55 years < age ≤ 65 years, age > 65 years. *Significant difference between genders with P < 0.01. (**B**) Prevalence of metabolic syndrome in different total bilirubin (TBIL) subgroups. TBIL subgroups 1 to 4 in male referred to the followings: TBIL ≤ 9.90 μmol/L, 9.90 μmol/L < TBIL ≤ 12.90 μmol/L, 12.90 μmol/L < TBIL ≤ 16.80 μmol/L, TBIL > 16.80 μmol/L. TBIL subgroups 1 to 4 in female referred to the followings: TBIL ≤ 8.00 μmol/L, 8.00 μmol/L < TBIL ≤ 10.30 μmol/L, 10.30 μmol/L < TBIL ≤ 13.40 μmol/L, TBIL > 13.40 μmol/L. *significant difference between genders with P < 0.01. (**C**) Prevalence of metabolic syndrome in different gamma glutamyltransferase (GGT) subgroups. GGT subgroups 1 to 4 in male referred to the followings: GGT ≤ 20.00U/L, 20.00U/L < GGT ≤ 30.00U/L, 30.00U/L < GGT ≤ 48.00U/L, GGT > 48.00U/L. GGT subgroups 1 to 4 in female referred to the followings: GGT ≤ 11.00U/L, 11.00U/L < GGT ≤ 15.00U/L, 15.00U/L < GGT ≤ 21.00U/L, GGT > 21.00U/L. *significant difference between genders with P < 0.01. (**D**) Prevalence of metabolic syndrome in different alanine aminotransferase (ALT) subgroups. ALT subgroups 1 to 4 in male referred to the followings: ALT ≤ 17.00U/L, 17.00U/L < ALT ≤ 24.00U/L, 24.00U/L < ALT ≤ 34.00U/L, ALT > 34.00U/L. ALT subgroups 1 to 4 in female referred to the followings: ALT ≤ 13.00U/L, 13.00U/L < ALT ≤ 16.00U/L, 16.00U/L < ALT ≤ 22.00U/L, ALT > 22.00U/L. *significant difference between genders with P < 0.01.

Overall prevalence of MS was 32.51% (10,654/32,768 cases) in our population. Males (37.72%, 7,786/20,643 cases) had significantly higher MS prevalence than females (23.65%, 2,868/12,125 cases), with a Chi-square value of 5578.583 (P < 0.01). Age had important influence on the prevalence of MS, rendering a crisscross pattern (Fig. 1A). In both genders, the prevalence of MS showed an increasing tendency from the youngest age to the age range of 45 to 55 years. Then from this age range to the highest age subgroup, the prevalence of MS reduced slowly in males, while a significantly sharp increase of MS prevalence was displayed in females. In brief, before menopause (younger than 45 years), male had significantly higher MS prevalence than females, after menopause (older than 65 years), females had significantly higher MS prevalence than males.

According to the various levels of TBIL, GGT and ALT, MS incidence demonstrated different patterns (Fig. 1B–D). First, males had a higher MS prevalence than females in all liver functional status. Second, MS prevalence presented an increasing trend with the rises of GGT and ALT levels, while MS prevalence presented a decreasing trend with the rise of TBIL.

### Correlations of key variables in different genders

Age demonstrated positive correlations with WC, SBP, DBP, LDL, HDL, TC, BUN, Cr and FG, yet negative correlations with GGT, ALT, TG and UA in males. In females, age demonstrated a negative correlation with HDL only, yet positive correlations with BMI, WC, SBP, DBP, TBIL, GGT, ALT, LDL, TC, TG, BUN, UA, Cr and FG.

TBIL showed positive correlations with GGT, ALT, HDL and Cr, yet negative correlations with BMI, WC, LDL, TC, TG, BUN, UA and FG in males. In females, TBIL showed positive correlations with age, ALT, HDL and Cr, yet negative correlations with BMI, WC, TG, BUN and FG.

GGT displayed positive correlations with BMI, WC, SBP, DBP, TBIL, ALT, LDL, TC, TG, UA and FG, yet negative correlations with age, BUN and Cr in males. In females, GGT displayed a negative correlation with HDL only, yet positive correlations with age, BMI, WC, SBP, DBP, ALT, LDL, TC, TG, BUN, UA and FG.

ALT had positive correlations with BMI, WC, SBP, DBP, TBIL, GGT, LDL, TC, TG, UA and FG, yet negative correlations with age, HDL, BUN and Cr in males. In females, ALT had negative correlations with HDL and Cr only, yet positive correlations with age, BMI, WC, SBP, DBP, TBIL, GGT, LDL, TC, TG, BUN, UA and FG (Table 5).

### Risks of developing MS in different genders

The risks of developing MS were calculated by four binary logistic regression models (Table 6). The first model designated TBIL quartiles as the categorical variables, and the lowest quartile was determined as reference. Age, BMI, GGT, ALT, BUN, UA and Cr were included as covariates. The protective effect against MS was demonstrated in both genders. The second model used GGT quartiles as the categorical variables, with the lowest quartile as reference. Age, BMI, TBIL, ALT, BUN, UA and Cr were covariates. Significantly increased risk was demonstrated in quartile 2 to 4 for both genders. The third model set ALT quartiles as the categorical variables. Age, BMI, TBIL, GGT, BUN, UA and Cr were covariates. Significantly increased risk was demonstrated in both genders as well. The last model had age as the categorical variable with the subgroup of age ≤25 years as reference. BMI, TBIL, GGT, ALT, BUN, UA and Cr were included as covariates. Significantly increased risk was demonstrated in age subgroups 2 to 6 for males, while in age subgroups 3 to 6 for females.

## Discussion

With the rapid economic growth and urbanization, China has been experiencing an epidemic of metabolic diseases. MS, defined as a constellation of interrelated metabolic abnormalities, is prevalent in China. MS prevalence has been reported to be from 6.6% to 25.6% in the Chinese population^8^,^9^,^15^,^16^, while the latest report showed that this figure jumped to 33.9%^3^. The key mechanisms of MS were related with insulin resistance, obesity, chronic low-grade systemic inflammation, endothelial dysfunction, etc. Actually, MS could be perceived as a pre-disease state of cardiovascular diseases, diabetes, fatty liver, chronic kidney disease or other chronic diseases^1^,^15^,^17^. There were obvious sex-specific, ethnicity-specific, and age-specific disparities in MS prevalence^18^. In fact, several previous studies showed a crisscross pattern of MS prevalence in different ages of men and women. In specific, young men have significantly higher MS prevalence than young women. After menopause, MS prevalence in women surpasses men^2^,^4^,^6^,^8^,^9^,^19^. The same results were also displayed in the current study. Gender differences were also demonstrated among other MS related topics^6^,^20^. For example, Song *et al*.^6^ revealed that a higher education level and a higher family income were associated with a higher MS prevalence in men, but associated with a lower MS prevalence in women. Higher physical activity was associated with a decreased MS prevalence in men, but associated with an increased MS prevalence in women. Compared with rice as the major staple food, cooked wheaten foods were associated with lower adjusted odds for MS in both genders. In a German study, Moebus *et al*.^20^ showed men without MS had an estimated mean 10-year risk of 4.7% for myocardial infarction, whereas the mean 10-year risk of men with MS was clearly higher (7.9%). In women without MS the mean 10-year risk for myocardial infarction was 1.1%, while in those with MS 2.3%. The alterations in sex hormones might be the main reason behind the above changes, in particular, a protective role of estrogen has been advocated^4^,^6^,^8^,^9^,^21^.

In our cross-sectional study, we documented significant associations of liver function markers (including TBIL, GGT and ALT) with MS after adjustment for age, BMI and kidney function. We showed that the prevalence of MS increased with higher quartiles of GGT and ALT in both genders, yet decreased with higher quartiles of TBIL. In other words, GGT and ALT were identified as risk factors for MS, while TBIL as a protective factor against MS. These associations between parameters of the liver function and MS have also been reported in a number of previous researches^11^,^16^,^22^,^23^,^24^,^25^,^26^. One point worth mentioning here was that for the majority of MS cases, the indices of liver function were within the normal ranges. Therefore, the prevalence of subclinical liver diseases and MS might be underestimated by the current normal reference range of the liver function. A proposal that upper normal limit of liver function should be lowered was recommended^26^,^27^, which could help improve the sensitivity in predicting MS and identify individuals at risk of MS much earlier. So, instead of using normal reference ranges, we used quartiles in the current investigation.

In our study, we showed negative correlations between TBIL and WC, TG, BP or FG, the latter indices were the key ingredients of MS. Furthermore, TBIL was proven as a protective factor against MS in both genders. It was described that MS was associated with insulin resistance, which was accompanied by oxidative stress. Oxidative stress was known to induce inflammatory mediators^28^. The results from two clinical investigations suggested that oxidative stress had a close association with abdominal obesity and hypertriglyceridemia, two components of MS^29^,^30^. It was logical to deduce that negative association between TBIL and abdominal obesity and hypertriglyceridemia could ensue the negative correlation between TBIL and MS^30^. Then, what could be the main reason for its protective effect against MS? There is evidence indicating that bilirubin can function as an antioxidant and cytoprotectant against oxidative stress by scavenging excess reactive oxygen species^31^,^32^. Besides, high sensitivity C-reactive protein was shown to have a negative association with bilirubin^33^. Therefore, as a potent endogenous antioxidant and cytoprotectant, the protective effect of TBIL against MS was related with not only the antioxidant effect, but also with its anti-inflammatory effect. Collectively, emerging evidence proved that the TBIL level was inversely associated with MS^19^,^30^,^34^. A recent meta-analysis from Nano *et al*.^19^ showed a pooled odd ratios (OR) for MS was 0.70 when comparing participants in the top versus bottom tertiles of bilirubin in fully adjusted regression models. Therefore, TBIL could be potentially employed as an early biomarker for indication of an increased risk of MS^35^.

MS and non-alcoholic fatty liver disease (NAFLD) can be generally considered as two definitions of the same risk of metabolic profiles^36^. The same prevalence pattern has been found in NAFLD, just like MS. NAFLD prevalence in men increases from younger to middle age and starts to decline after the age of 50–60 years, forming an “inverted U shaped curve”. Conversely, NAFLD prevalence in women increased after the age of 50, peaking at 60–69 years and declining after the 7th decade of life^37^. And, as a pathogenic determinant of MS, NAFLD often coexisted with all features of MS^1^,^38^,^39^,^40^,^41^. In fact, the latest researches suggested that there was a complex relationship between NAFLD and MS^36^,^42^,^43^,^44^. In an Italian study by Lonardo *et al*.^43^, NAFLD was perceived as a precursor of MS. This study showed most individuals with NAFLD had insulin resistance, but only a minority of those with NAFLD exhibited the full blown MS, NAFLD was most likely a precursor and a necessary prerequisite to the development of progressive metabolic disease. However, in another study by Yki-Jarvinen^36^, NAFLD was considered as not only a cause, but also a consequence of MS. In a recent meta-analysis, Ballestri *et al*.^44^ analyzed a pooled population of 81,411 patients who were followed-up for a median period of 4.5 years, NAFLD was associated with an increased risk of incident MS with a pooled OR of 1.98 for GGT and 1.80 for ALT, respectively. The current study demonstrated a close positive relationship between elevated liver transaminase (GGT and ALT) and MS risk. The possible physiological mechanisms that lead to such a phenomenon exists in oxidative stress and insulin resistance as well^36^,^41^,^45^. As a matter of fact, NAFLD is defined as the presence of fat accumulation in liver exceeding 5% of hepatocytes, in the absence of excessive alcohol intake, viral infection, or any other specific etiology of liver disease. Clear evidence implies that lipid droplet accumulation in liver decrease the efficiency of insulin signaling producing resistance and the effectiveness of insulin to signal within the cell to maintain tissue homeostasis^36^,^39^,^42^.

There were several shortcomings in our work deserving some comments. First, as a cross-sectional survey, we could not determine the causality relationship. Therefore, a study with prospective nature is warranted in the future. Second, because of the budget shortage, we did not measure parameters of oxidative stress, mediators of inflammation and sex hormones of the participants in our study. Third, the blood parameters were checked only once because of budget shortage as well. Fourth, although the exclusion criteria was applied strictly to eliminate those with diseases which might influence the levels of liver function, the medical conditions of some ostensibly healthy participants might not be known before hand, which might be a confounding factor in our study.

In conclusion, this study determined that the levels of liver function were indeed associated with the MS risks, and gender and age had great impacts on the association. A high TBIL level showed protective effect against MS, while high GGT and ALT levels were risk factors for MS. It is necessary that liver function should be seriously evaluated to judge the MS risk in individuals.

## Methods

### Design

We conducted this cross-sectional, community-based health-check investigation in Tianjin Medical University General Hospital, under cooperation from the departments of Health Management, Ultrasound, and Nuclear Medicine. During the period from September 2011 through March 2016, a total of 32,768 subjects (20,643 male, 12,125 female) who self-reported as healthy had adequate data for analysis. After completing a questionnaire, all participants were asked to provide a blood sample and receive an overall heath check. In order to avoid the influence of confounding factors, the following criteria were used for exclusion: subjects with disease history of liver diseases; subjects with any diseases or taking any medicine that might affect liver function; subjects during pregnancy and subjects with malignancy.

### Ethics

The institutional review board and ethic committee of Tianjin Medical University General Hospital approved the ethical, methodological and protocol aspects of this investigation. We confirm that all methods in the current study were carried out in accordance with the relevant guidelines and regulations. All participants in this research provided their written consents.

### Measurements

After the participants visited our institution, anthropometric measurements and fasting blood tests of the participants were performed. Body height (BH) and body weight (BW) were measured in centimeters and kilograms. BMI was calculated by dividing BW (kilograms) by the square of BH (meters^2^). TBIL, ALT, LDL, HDL, TC, TG, BUN, UA, Cr and FG were measured by an auto-analyzer (Hitachi Model 7600 analyzer, Hitachi, Tokyo, Japan). Ultrasonography (MyLab Classs C, Technos, Esaote Biomedica, Genoa, Italy) on the abdomen and heart was performed in each individual.

The laboratory reference ranges for parameters were as follows: TBIL 3.40–20.00 μmol/L; GGT 7.00–49.00U/L; ALT 5.00–40.00U/L; LDL 1.33–3.36 mmol/L; HDL 0.80–2.20 mmol/L TC 3.59–5.17 mmol/L; TG 0.57–1.71 mmol/L; BUN 1.70–8.30 mmol/L; UA 140.00–414.00 μmol/L; Cr 44.00–115.00 μmol/L; FG 3.60–5.80 mmol/L.

### Definition

MS was diagnosed when at least three of the following criteria were met^1^,^8^,^9^: (1) WC ≥ 90 cm in men, ≥ 80 cm in women (2) TG ≥ 1.70 mmol/L, (3) HDL < 1.03 mmol/L in men, <1.29 mmol/L in women, (4) SBP ≥ 130 mmHg or DBP ≥ 85 mmHg, and (5) FG ≥ 5.60 mmol/L.

TBIL, GGT and ALT were divided based on quartiles of the measurements. Age subgroups 1 to 6 were formulated according to the following respectively: age ≤ 25 years, 25 years < age ≤ 35 years, 35 years < age ≤ 45 years, 45 years < age ≤ 55 years, 55 years < age ≤ 65 years, age > 65 years.

### Statistical analysis

All data were represented as mean ± standard deviation. Independent sample’s t test was used to compare the differences of indices between groups or subgroups. The inter-group differences were analyzed by Chi-square test. Pearson bivariate correlation was made among variables. OR for MS with 95% CI were calculated by binary logistic regression models. Statistical Package for Social Sciences (SPSS version 17.0, Chicago, IL, USA) was used to conduct statistics and significance was defined as P < 0.05.

## Additional Information

**How to cite this article:** Wang, S. *et al*. Association between liver function and metabolic syndrome in Chinese men and women. *Sci. Rep.* **7**, 44844; doi: 10.1038/srep44844 (2017).

**Publisher's note:** Springer Nature remains neutral with regard to jurisdictional claims in published maps and institutional affiliations.

## Figures and Tables

**Table 1:** Population characteristics based on different genders.

	Total	Male	Female	T value
Case number	32768	20643	12125	
Age (years)	48.65 ± 11.05	48.47 ± 10.83	48.95 ± 11.42	−3.739**
BMI (kg/m2)	25.48 ± 3.46	26.15 ± 3.25	24.35 ± 3.52	46.724**
WC (cm)	86.93 ± 10.69	90.94 ± 9.05	80.10 ± 9.75	101.801**
SBP (mmHg)	124.51 ± 17.90	126.02 ± 16.69	121.95 ± 19.51	20.014**
DBP (mmHg)	79.28 ± 11.69	81.63 ± 11.52	75.28 ± 10.87	49.134**
TBIL (μmol/L)	12.94 ± 6.05	13.97 ± 6.48	11.19 ± 4.75	41.277**
GGT (U/L)	33.91 ± 41.41	42.03 ± 47.57	20.11 ± 21.90	47.848**
ALT (U/L)	25.45 ± 19.47	29.00 ± 21.59	19.41 ± 13.17	44.313**
LDL (mmol/L)	3.11 ± 0.87	3.11 ± 0.85	3.10 ± 0.90	0.953
HDL (mmol/L)	1.37 ± 0.36	1.27 ± 0.32	1.54 ± 0.36	−68.496**
TC (mmol/L)	5.20 ± 0.99	5.18 ± 0.97	5.24 ± 1.02	−5.156**
TG (mmol/L)	1.75 ± 1.39	1.98 ± 1.55	1.36 ± 0.97	39.594**
BUN (mmol/L)	4.81 ± 1.30	5.04 ± 1.30	4.45 ± 1.22	40.971**
UA (μmol/L)	329.29 ± 86.52	367.08 ± 76.51	264.94 ± 60.76	125.571**
Cr (μmol/L)	72.11 ± 16.62	79.44 ± 15.28	59.64 ± 10.09	127.303**
FG (mmol/L)	5.28 ± 1.23	5.40 ± 1.34	5.06 ± 0.98	24.280**

BMI = body mass index, WC = waist circumference, SBP = systolic blood pressure, DBP = diastolic blood pressure, TBIL = total bilirubin, GGT = gamma glutamyltransferase, ALT = alanine aminotransferase, LDL = low-density lipoprotein cholesterol, HDL = high-density lipoprotein cholesterol, TC = total cholesterol, TG = triglycerides, BUN = blood urea nitrogen, UA = uric acid, Cr = creatinine, FG = fasting glucose. **P < 0.01 (analyzed by independent sample’s t test).

**Table 2:** Percentage distribution of TBIL quartiles in different genders.

	Percentage distribution (and case number count) in different age subgroups (years)						
	Age ≤ 25	25 < age ≤ 35	35 < age ≤ 45	45 < age ≤ 55	55 < age ≤ 65	Age > 65	Total
Male (μmol/L)							
TBIL ≤ 9.90	24.59%(30)	25.89%(502)	26.14%(1738)	25.37%(1752)	24.01%(863)	21.30%(305)	25.14%(5190)
9.90 < TBIL ≤ 12.90	19.67%(24)	24.19%(469)	25.25%(1679)	25.25%(1744)	25.63%(921)	25.91%(371)	25.23%(5208)
12.90 < TBIL ≤ 16.80	22.13%(27)	23.47%(455)	23.67%(1574)	25.53%(1763)	26.38%(948)	26.40%(378)	24.92%(5145)
TBIL > 16.80	33.61%(41)	26.46%(513)	24.95%(1659)	23.85%(1647)	23.98%(862)	26.40%(378)	24.71%(5100)
Female (μmol/L)							
TBIL ≤ 8.00 μmol/L	33.81%(47)	25.68%(291)	28.72%(1075)	26.30%(992)	22.70%(518)	18.84%(199)	25.75%(3122)
8.00 < TBIL ≤ 10.30	22.30%(31)	23.83%(270)	23.19%(868)	25.69%(969)	27.34%(624)	26.52%(280)	25.09%(3042)
10.30 < TBIL ≤ 13.40	17.27%(24)	24.45%(277)	24.10%(902)	24.10%(909)	24.58%(561)	27.75%(293)	24.46%(2966)
TBIL > 13.40	26.62%(37)	26.04%(295)	23.99%(898)	23.91%(902)	25.37%(579)	26.89%(284)	24.70%(2995)
Chi-square value^							
Total	3.935	0.387	11.127*	3.001	5.436	2.360	1.827

TBIL = total bilirubin. ^Comparing the percentage distribution between males and females by Chi-square method. *P < 0.05.

**Table 3:** Percentage distribution of GGT quartiles in different genders.

	Percentage distribution (and case number count) in different age subgroups (years)						
	Age ≤ 25	25 < age ≤ 35	35 < age ≤ 45	45 < age ≤ 55	55 < age ≤ 65	Age > 65	Total
Male (U/L)							
GGT ≤ 20.00	54.10%(66)	34.66%(672)	22.39%(1489)	21.24%(1467)	28.63%(1029)	48.39%(693)	26.24%(5416)
20.00 < GGT ≤ 30.00	23.77%(29)	22.90%(444)	23.73%(1578)	25.63%(1770)	29.24%(1051)	28.42%(407)	25.57%(5279)
30.00 < GGT ≤ 48.00	13.93%(17)	19.08%(370)	24.41%(1623)	26.11%(1803)	25.13%(903)	15.64%(224)	23.93%(4940)
GGT > 48.00	8.20%(10)	23.36%(453)	29.47%(1960)	27.02%(1866)	17.00%(611)	7.54%(108)	24.26%(5008)
Female (U/L)							
GGT ≤ 11.00	53.24%(74)	45.19%(512)	35.96%(1346)	24.10%(909)	14.20%(324)	17.14%(181)	27.60%(3346)
11.00 < GGT ≤ 15.00	26.62%(37)	28.86%(327)	28.40%(1063)	23.99%(905)	23.05%(526)	27.18%(287)	25.94%(3145)
15.00 < GGT ≤ 21.00	12.95%(18)	16.15%(183)	18.76%(702)	22.72%(857)	27.08%(618)	25.28%(267)	21.81%(2645)
GGT > 21.00	7.19%(10)	9.80%(111)	16.88%(632)	29.19%(1101)	35.67%(814)	30.40%(321)	24.65%(2989)
Chi-square value^							
Total	0.350	105.808**	368.597**	26.923**	348.899**	382.110**	20.933**

GGT = gamma glutamyltransferase. ^Comparing the percentage distribution between males and females by Chi-square method. **P < 0.01.

**Table 4:** Percentage distribution of ALT quartiles in different genders.

	Percentage distribution (and case number count) in different age subgroups (years)						
	Age ≤ 25	25 < age ≤ 35	35 < age ≤ 45	45 < age ≤ 55	55 < age ≤ 65	Age > 65	Total
Male (U/L)							
ALT ≤ 17.00	39.34%(48)	23.16%(449)	19.91%(1324)	22.30%(1540)	31.69%(1139)	46.37%(664)	25.02%(5164)
17.00 < ALT ≤ 24.00	26.23%(32)	23.78%(461)	24.75%(1646)	28.08%(1939)	32.55%(1170)	30.45%(436)	27.53%(5684)
24.00 < ALT ≤ 34.00	18.85%(23)	19.70%(382)	23.94%(1592)	25.38%(1753)	22.18%(797)	15.43%(221)	23.10%(4768)
ALT > 34.00	15.57%(19)	33.37%(647)	31.40%(2088)	24.24%(1674)	13.58%(488)	7.75%(111)	24.35%(5027)
Female (U/L)							
ALT ≤ 13.00	57.55%(80)	49.60%(562)	42.19%(1579)	25.72%(970)	18.89%(431)	27.84%(294)	32.30%(3916)
13.00 < ALT ≤ 16.00	17.27%(24)	18.71%(212)	20.22%(757)	19.33%(729)	19.24%(439)	20.17%(213)	19.58%(2374)
16.00 < ALT ≤ 22.00	18.71%(26)	18.36%(208)	21.85%(818)	27.09%(1022)	29.89%(682)	28.50%(301)	25.21%(3057)
ALT > 22.00	6.47%(9)	13.33%(151)	15.74%(589)	27.86%(1051)	31.99%(730)	23.48%(248)	22.91%(2778)
Chi-square value^							
Total	11.841**	271.588**	679.275**	102.171**	437.263**	232.556**	363.689**

ALT = alanine aminotransferase. ^^^Comparing the percentage distribution between males and females by Chi-square method. **P < 0.01.

**Table 5:** Pearson bivariate correlations among key variables based on different genders.

	Correlation coefficients for males				Correlation coefficients for females			
	age	TBIL	GGT	ALT	age	TBIL	GGT	ALT
age	—	0.005	−0.073**	−0.171**	—	0.024**	0.157**	0.140**
BMI	−0.012	−0.068**	0.145**	0.259**	0.302**	−0.065**	0.161**	0.258**
WC	0.120**	−0.076**	0.170**	0.238**	0.445**	−0.053**	0.175**	0.251**
SBP	0.295**	−0.005	0.118**	0.070**	0.546**	−0.009	0.147**	0.148**
DBP	0.102**	0.002	0.173**	0.123**	0.309**	−0.003	0.129**	0.148**
TBIL	0.005	—	0.099**	0.045**	0.024**	—	0.015	0.043**
GGT	−0.073**	0.099**	—	0.387**	0.157**	0.015	—	0.448**
ALT	−0.171**	0.045**	0.387**	—	0.140**	0.043**	0.448**	—
LDL	0.082**	−0.071**	0.078**	0.064**	0.319**	−0.016	0.154**	0.111**
HDL	0.056**	0.094**	0.006	−0.092**	−0.037**	0.056**	−0.054**	−0.071**
TC	0.071**	−0.034**	0.199**	0.115**	0.371**	−0.004	0.191**	0.146**
TG	−0.074**	−0.088**	0.275**	0.185**	0.237**	−0.067**	0.188**	0.190**
BUN	0.175**	−0.071**	−0.030**	−0.033**	0.292**	−0.040**	0.060**	0.049**
UA	−0.097**	−0.038**	0.151**	0.153**	0.221**	0.009	0.183**	0.176**
Cr	0.059**	0.027**	−0.060**	−0.046**	0.162**	0.023*	0.016	−0.024**
FG	0.146**	−0.031**	0.150**	0.079**	0.278**	−0.060**	0.163**	0.139**

BMI = body mass index, WC = waist circumference, SBP = systolic blood pressure, DBP = diastolic blood pressure, TBIL = total bilirubin, GGT = gamma glutamyltransferase, ALT = alanine aminotransferase, LDL = low-density lipoprotein cholesterol, HDL = high-density lipoprotein cholesterol, TC = total cholesterol, TG = triglycerides, BUN = blood urea nitrogen, UA = uric acid, Cr = creatinine, FG = fasting glucose. *P < 0.05, **P < 0.01.

**Table 6:** The likelihood of MS in different genders.

	Males		Females	
	Parameter values	OR (CI)	Parameter values	OR (CI)
TBIL quartiles &	(μmol/L)			
Quartile 1	TBIL ≤ 9.90 (reference)		TBIL ≤ 8.00 (reference)	
Quartile 2	9.90 < TBIL ≤ 12.90	0.829 (0.758–0.908)**	8.00 < TBIL ≤ 10.30	0.874 (0.762–1.001)
Quartile 3	12.90 < TBIL ≤ 16.80	0.814 (0.743–0.891)**	10.30 < TBIL ≤ 13.40	0.761 (0.661–0.875)**
Quartile 4	TBIL > 16.80	0.673 (0.613–0.739)**	TBIL > 13.40	0.753 (0.653–0.867)**
GGT quartiles $	(U/L)			
Quartile 1	GGT ≤ 20.00 (reference)		GGT ≤ 11.00 (reference)	
Quartile 2	20.00 < GGT ≤ 30.00	1.710 (1.546–1.892)**	11.00 < GGT ≤ 15.00	1.730 (1.458–2.052)**
Quartile 3	30.00 < GGT ≤ 48.00	2.438 (2.202–2.700)**	15.00 < GGT ≤ 21.00	2.425 (2.048–2.871)**
Quartile 4	GGT > 48.00	3.715 (3.335–4.138)**	GGT > 21.00	3.027 (2.552–3.592)**
ALT quartiles #	(U/L)			
Quartile 1	ALT ≤ 17.00 (reference)		ALT ≤ 13.00 (reference)	
Quartile 2	17.00 < ALT ≤ 24.00	1.341 (1.217–1.477)**	13.00 < ALT ≤ 16.00	1.145 (0.979–1.339)
Quartile 3	24.00 < ALT ≤ 34.00	1.594 (1.441–1.764)**	16.00 < ALT ≤ 22.00	1.225 (1.062–1.413)**
Quartile 4	ALT > 34.00	2.068 (1.858–2.302)**	ALT > 22.00	1.612 (1.390–1.870)**
Age subgroups ^	(years)			
Age subgroup 1	Age ≤ 25 (reference)		Age ≤ 25 (reference)	
Age subgroup 2	25 < Age ≤ 35	2.224 (1.068–4.630)*	25 < Age ≤ 35	2.105 (0.696–6.372)
Age subgroup 3	35 < Age ≤ 45	4.614 (2.235–9.529)**	35 < Age ≤ 45	3.249 (1.101–9.589)*
Age subgroup 4	45 < Age ≤ 55	6.784 (3.285–14.011)**	45 < Age ≤ 55	6.855 (2.329–20.179)**
Age subgroup 5	55 < Age ≤ 65	7.585 (3.665–15.701)**	55 < Age ≤ 65	13.497 (4.579–39.785)**
Age subgroup 6	Age > 65	9.357 (4.489–19.501)**	Age > 65	18.031 (6.085–53.429)**

MS = metabolic syndrome, OR = odds ratio, CI = confidence interval, BMI = body mass index, TBIL = total bilirubin, GGT = gamma glutamyltransferase, ALT = alanine aminotransferase, BUN = blood urea nitrogen, UA = uric acid, Cr = creatinine. ^&^Logistic regression model included age, BMI, GGT, ALT, BUN, UA and Cr as covariates. ^$^Logistic regression model included age, BMI, TBIL, ALT, BUN, UA and Cr as covariates. ^#^Logistic regression model included age, BMI, TBIL, GGT, BUN, UA and Cr as covariates. ^^^Logistic regression model included BMI, TBIL, GGT, ALT, BUN, UA and Cr as covariates. *P < 0.05, **P < 0.01.
